# Comparison of Self-Etching Adhesives and Etch-and-Rinse Adhesives on the Failure Rate of Posterior Composite Resin Restorations: A Systematic Review and Meta-Analysis

**DOI:** 10.1055/s-0041-1736332

**Published:** 2021-11-22

**Authors:** Basílio Rodrigues Vieira, Eugênia Lívia de Andrade Dantas, Yuri Wanderley Cavalcanti, Bianca Marques Santiago, Frederico Barbosa de Sousa

**Affiliations:** 1 Graduate Program in Dentistry, Health Sciences Center, Federal University of Paraíba, João Pessoa, Paraiba, Brazil; 2 Department of Clinical and Social Dentistry, Health Sciences Center, Federal University of Paraíba, João Pessoa, Paraiba, Brazil; 3 Department of Morphology, Health Sciences Center, Federal University of Paraíba, João Pessoa, Paraiba, Brazil

**Keywords:** dentine adhesives, resin composite, dental restoration, failures, clinical studies, meta-analysis

## Abstract

The aim of this study was to perform a systematic review with meta-analysis on the comparison of self-etching adhesives and etch-and-rinse adhesives with respect to the failure rate of posterior composite resin restorations. The study protocol was registered in PROSPERO (CRD42017078015), following PRISMA recommendations and PICO search strategy. Literature search was performed in the following databases: MEDLINE, ISI Web of Science, LILACS, SCOPUS, and Cochrane Library through July 2021. Six studies from five randomized clinical trials were included in the qualitative synthesis. The funnel plot detected important bias (all studies out of the funnel area). The meta-analysis showed a positive summary Cohen H effect size of 0.406 (95% CI: 0.100; 0.713, *p* = 0.009), favoring etch-and-rinse adhesives. The total number of failures (including restorations that required replacement and those that did not require replacement) were attributed to either marginal adaptation (five studies) or marginal staining (one study). A very low certainty of the evidence was obtained through GRADE analysis. In conclusion, current available evidence indicates that etch-and rinse adhesives performed better (with a low effect size) than self-etching adhesives in terms of failure rates in posterior composite restorations.

## Introduction

Dentine adhesives, which have undergone substantial changes over the last 20 years, are classified into two techniques: self-etch or etch-and-rinse. 1 Etch-and-rinse, the first to be introduced, is the technique that results in the deepest hybrid layer in enamel. 2 Because of the higher number of steps and stronger effect of the etching procedure on dentine substrate, etch-and-rinse technique requires a longer clinical application time, results in increased postoperative sensitivity, and is the more sensitive to failure. 2

The shorter application time and decreased postoperative sensitivity favors the choice for the self-etch, 3 but their thinner hybrid layer raises concern on whether the durability of the restoration is reduced or not. 4 At the other hand, the thinner dentine hybrid layer theoretically provides less substrate to be degraded by chemical (both hydrolysis and enzymatic) and mechanical factors.

Adhesive composite restorations in posterior teeth are currently the first choice for direct restorations in posterior teeth, 5 6 and their popularity is expected to increase with current prevailing conservative philosophy in the restorative treatment where minimal hard dental tissue removal is recommended. Self-etch technique is in line with such a philosophy, and short duration clinical studies have reported similarities between self-etch and etch-and-rinse techniques with respect to clinical outcomes. 7 8 9 However, relying mostly on statistical analysis restricted to *p* -values (statistical significance), the individual scientific contribution of those studies to the choice of the appropriate dentine adhesive technique is questionable. 10 11

To the best of our knowledge, there are no systematic reviews with meta-analysis comparing self-etch and etch-and-rinse techniques for dentine adhesives in posterior composite restorations. Filling the gap in such an important topic in clinical Dentistry could provide an important contribution to the restorative dental practice with maximum preservation of tooth structure.

Therefore, the aim of this study was to perform a systematic review with meta-analysis on the comparison of self-etching adhesives and etch-and-rinse adhesives on the failure rate of posterior composite resin restorations.

## Methods

### Focused Question

This systematic review was aimed at answering the following research question: do composite resin restorations in posterior teeth performed with either self-etch or etch-and-rinse techniques differ in the clinical failure rate?

This review followed the PRISMA guidelines, 12 and its protocol was registered in the *International Prospective Register of Systematic Reviews* (PROSPERO) under the number CRD42017078015.

### Search Strategy

The literature search included studies published through July 2021, and it was undertaken by two independent researchers in the following databases: MEDLINE (PubMed), ISI Web of Science, LILACS, SCOPUS, and Cochrane Library, in addition to searches in grey literature (Google Scholar and manual search in the list of references of included studies).

Search strategies for the literature are based on PICO acronym. 13 A combination of MeSH terms, keywords, and related terms was used in the systematic literature search in conjunction with Boolean operators “AND” and “OR” ( Table 1 ).

**Table 1:** Search strategies for the literature based on PICO acronym, with the use of Boolean operators and adapted to each database

Databases	Search strategies
**PubMed**	(((((((Self-etching adhesives[Title/Abstract]) OR Self etching adhesive[Title/Abstract]) OR All in one adhesive[Title/Abstract]) OR All in one adhesives[Title/Abstract]) OR One-step adhesive[Title/Abstract])) AND (((((((((((((Dentin-bonding agents[MeSH Terms]) OR Dentin-bonding agents[Title/Abstract]) OR Agents, Dentin-Bonding[Title/Abstract]) OR Bonding Agents, Dentin[Title/Abstract]) OR Agents, Dentin Bonding[Title/Abstract]) OR Dentin Bonding Agents[Title/Abstract]) OR Etch[Title/Abstract]) OR rinse adhesives[Title/Abstract]) OR Etch-and-rinse[Title/Abstract]) OR Three step adhesive[Title/Abstract]) OR Three step adhesives[Title/Abstract])))) AND (((((((Dental Restoration Failure[MeSH Terms]) OR Dental Restoration Failure[Title/Abstract]) OR Failure, Dental Restoration[Title/Abstract]) OR Restoration Failure, Dental) OR Restoration Failures, Dental) OR Dental Restoration Failures) OR Failures, Dental Restoration)
**Scopus**	(TITLE-ABS-KEY(Self-etching adhesives) OR TITLE-ABS-KEY(Self etching adhesive) OR TITLE-ABS-KEY(All in one adhesive) OR TITLE-ABS-KEY(All in one adhesives) OR TITLE-ABS-KEY (One-step adhesive)) AND (TITLE-ABS-KEY(Dentin-bonding agents) OR TITLE-ABS-KEY(Agents, Dentin-Bonding) OR TITLE-ABS-KEY(Bonding Agents, Dentin) OR TITLE-ABS-KEY(Agents, Dentin Bonding) OR TITLE-ABS-KEY (Etch) TITLE-ABS-KEY(rinse adhesives) OR TITLE-ABS-KEY(Etch-and-rinse) OR TITLE-ABS-KEY(Three step adhesive) OR TITLE-ABS-KEY(Three step adhesives)) AND (TITLE-ABS-KEY(Dental Restoration Failure) OR TITLE-ABS-KEY(Failure, Dental Restoration) OR TITLE-ABS-KEY(Failure, Dental Restoration) OR TITLE-ABS-KEY(Restoration Failures, Dental) OR TITLE-ABS-KEY (Dental Restoration Failures) OR TITLE-ABS-KEY (Failures, Dental Restoration))
**Lilacs**	((TW:(Self-etching adhesives)) OR (TW:(Self etching adhesive)) OR (TW:(All in one adhesive)) OR (TW:(All in one adhesives)) OR (TW:(One-step adhesive)) OR (TW:(Adesivo autocondicionantes)) OR (TW:(Adesivos autocondicionantes)) OR (TW:(Adesivo de passo único)) OR (TW:(Adesivos de passo único)) OR (TW:(Adesivo de um passo)) OR (TW:(Adhesivo autocondicionante)) OR (TW:(Adhesivos autocondicionantes)) OR (TW:(adhesivo de paso único)) OR (TW:(adhesivos de paso único)) OR (TW:(adhesivo de paso))) AND ((MH:(Dentin-bonding agents)) OR (TW:(Dentin-bonding agents)) OR (TW:(Agents, Dentin-Bonding)) OR (TW:(Bonding Agents, Dentin)) OR (TW:(Agents, Dentin Bonding)) OR (TW:(Dentin Bonding Agents)) OR (TW:(etch rinse adhesives)) OR (TW:(Etch-and-rinse)) OR (TW:(Three step adhesive)) OR (TW:(Three step adhesives)) OR (MH:(Adesivos dentinários)) OR (TW:(Adesivos dentinários)) OR (TW:(Agente de ligação a dentina)) OR (TW:(Agentes de ligações a dentina)) OR (TW:(Agente de união a dentina)) OR (TW:(Agentes de união a dentina)) OR (TW:(Adesivo convencional)) OR (TW:(Adesivos convencionais)) OR (TW:(Adesivo de três passos)) OR (TW:(Adesivos de três passos)) OR (MH:(Recubrimientos dentinarios)) OR (TW:(Recubrimientos dentinarios)) OR (TW:(Agente de unión a la dentina)) OR (TW:(Agentes de unión a la dentina)) OR (TW: (Agente de unión a dentina)) OR (TW:(Agentes de unión a dentina)) OR (TW:(Adhesivo convencional)) OR (TW:(adhesivos convencionales)) OR (TW:(Adhesivo de tres pasos)) OR (TW:(Adhesivos de tres pasos))) AND ((MH:(Dental Restoration Failure)) OR (TW:(Dental Restoration Failure)) OR (TW:(Failure, Dental Restoration)) OR (TW:(Restoration Failure, Dental)) OR (TW:(Restoration Failures, Dental)) OR (TW:(Dental Restoration Failures)) OR (TW:(Failures, Dental Restoration)) OR (MH:(Falha de restauração dentária)) OR (TW:(Falha de restauração dentária)) OR (TW:(Falha, Restauração dentária)) OR (TW:(Falha na restauração, dental)) OR (TW:(Falhas de Restaurações, dental)) OR (TW:(Falhas de restauração dentária)) OR (TW:(Falhas, Restaurações dentárias)) OR (MH:(Fracaso de la Restauración Dental)) OR (TW:(Fracaso de la Restauración Dental)) OR (TW:(Fracaso, Restauración Dental)) OR (TW:(Fracaso de la Restauración, dental)) OR (TW:(Fracasos de las Restauraciones, dental)) OR (TW:(Falla, restauración dental)) OR (TW:(Fallas, restauraciones dental)))
**Web of Science**	TS = (Self-etching adhesives OR Self etching adhesive OR All in one adhesive OR All in one adhesives OR One-step adhesive) AND TS = (Dentin-bonding agents OR Agents, Dentin-Bonding OR Bonding Agents, Dentin OR Agents, Dentin Bonding OR Dentin Bonding Agents OR etch rinse adhesives OR Etch-and-rinse OR Three step adhesive OR Three step adhesives) AND TS = (Dental Restoration Failure OR Failure, Dental Restoration OR Restoration Failure, Dental Restoration Failures, Dental OR Dental Restoration Failures OR Failures, Dental Restoration)

### Screening and Study Selection

Duplicate removal was undertaken by two independent examiners (B.R.V. and E.L.A.D.), using Mendeley software (version 1.5.2 for Windows). Article selection for inclusion was based on the evaluation of titles, abstracts (step1), and then evaluation of full texts (step 2). Only randomized clinical trials, controlled clinical trials, and nonrandomized controlled prospective studies were selected for this systematic review. Observational studies, case reports, cases series, *in vitro* studies, literature review, editorials, and letters to the editor were excluded.

Full analysis of selected articles was undertaken based on the following PICO terms 13 : Population represented by posterior permanent teeth with Class I or Class II resin composite restorations due to caries, Intervention represented by self-etch adhesives, Control represented by etch-and-rinse (conventional) adhesives, and Outcome represented by failures in restorations that compromise longevity. Disagreements between examiners were solved by consensus. When disagreement persisted, the opinion of a third examiner (B.M.S.) was used.

### Data Collection

Full texts were accessed for validation of eligibility criteria, and the following data were collected: study design, population, group sample, adhesive type, outcome, evaluation criteria, time of follow-up evaluation, statistical analysis, main results, failure rate (marginal staining, marginal adaptation, secondary caries, fractures and retention, and postoperative sensitivity), limitations, and conclusions. This was undertaken independently by two reviewers.

### Risk of Bias (Quality Assessment)

Quality assessment of selected studies was performed individually and independently by two examiners (B.R.V. and E.L.A.D.) using the *Cochrane Collaboration Risk of Bias tool* , 14 and the following aspects were analyzed: sequence generation, allocation concealment, blinding of participants and personnel, blinding of outcome assessors, incomplete outcome data, and other sources of bias. Studies were then classified as low, medium, or high risk of bias; those with insufficient information were classified as unclear.

### Data Analysis

From each study, differences between two groups (etch-and-rinse as control and self-etching as intervention) were considered. Using data on failure rates (proportions) and sample size per group for each study, we calculated the effect size of difference between proportions using Cohen H effect size [difference between arcsine transformation of proportions: arcsine * sqrt(p1) – arcsine * sqrt(p2)] and statistical power, following equations described in the literature. 15 Only failures related to the adhesive were included, which comprised marginal staining, marginal adaptation, secondary caries, fractures and retention, and postoperative sensitivity. The unit restoration with failure was considered as a restoration with one or more failures, so that computation of more than one failure per restoration was excluded. The unit restoration with failure was recorded regardless of the need of restoration replacement. For each group (intervention or control), proportions of restorations with failures were computed using the number of restorations with failures divided by the number of restorations.

A two-tailed 5% significance level was used. The 95% confidence interval (CI) for Cohen H was calculated using formula for sampling variance described elsewhere. 16 Considering that some failure rates in controls were lower than 10%, risk ratio (attributable risk) was not computed because it overestimates the effect size when the proportion of controls is lower than 10%. 17

Statistical power, whose threshold of 80% is used to determine whether studies were conclusive (acceptable probability that an effect exists in the population) 15 or not, was calculated for all studies selected for meta-analysis.

One meta-analysis was performed. Following published statistical procedures, 15 16 18 we calculated the effect size (Cohen H; es), standard error, sampling variance, individual study weights ( *w* ), the weighted effect sizes ( *w* * es), and the corresponding squared values ( *w* 2 and *w* * es2). Both Cochran *Q* test and *I* ^2^ were computed, 18 and the level of heterogeneity was graded as low (25%), moderate (50%), or high (75%). 19 The summary outcome was calculated using the fixed effects model when heterogeneity was very low, otherwise the random effects model was used. 19 The statistical power of the meta-analysis was also computed. 20 21 A forest plot was prepared using calculated parameters. In addition, bias was also investigated using funnel plots (scatter plots of effect sizes in the X axis against the effect size's standard error in the Y axis). 22

### Certainty of Evidence

The certainty of the evidence was assessed through Grades of Recommendations, Assessment, Development and Evaluation (GRADE) approach. The initial ratings followed the recommendations of GRADE group and the certainty of evidenced initiated as high, since this systematic review was performed with randomized clinical trials. The outcome “failure rate of resin restorations” was carefully analyzed for each of the five domains that can lower the certainty: risk of bias, inconsistency, indirectness, imprecision, and publication bias. 22

## Results

**Fig. 1:**
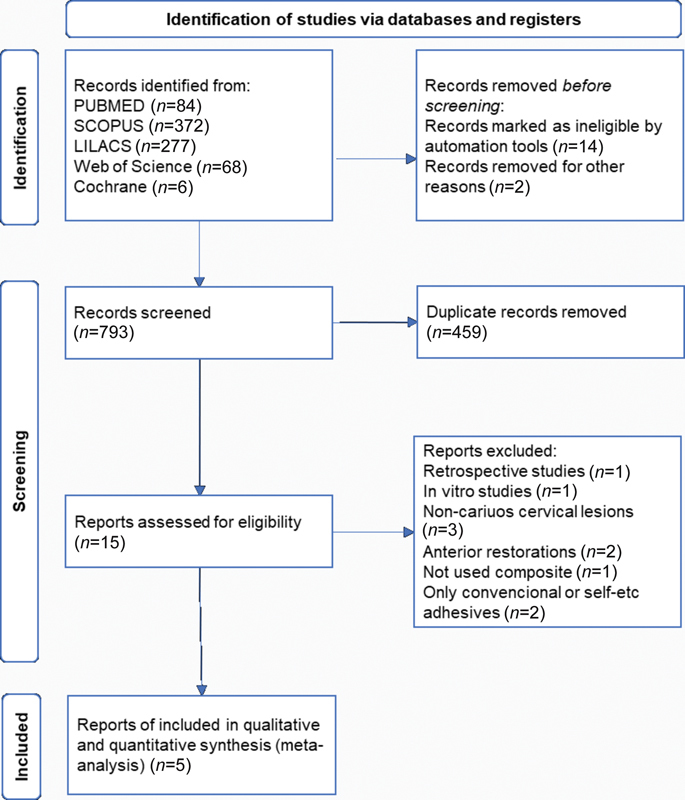
Flowchart of the steps of the literature search.

The flowchart and reasons for exclusion of articles are shown in Fig. 1 . A total of 823 articles were recovered, of which 459 were duplicates (removed using the Mendeley software). After careful analysis of titles and abstracts, 15 articles were selected for full text reading, and five articles 9 23 24 25 26 met the inclusion criteria. One article 16 contained three studies, and one study was excluded because the adhesive (iBond) was not recommended for clinical use by the authors, yielding a total of six studies (five papers with one study each and one paper with two studies) included in the review. The characteristics of the included studies are presented in Table 2 .

**Table 2:** Summary of data extracted from selected studies

Study (design)	Groups	Evaluation criteria - outcome	Number of failures (%)	Limitations	Conclusion (as reported in the paper)
Van Dijken and Pallesen (2017) 24 (Randomized Clinical Trial)	*N* = 114 C: Two-step etch-and-rise, Optibond ( *n* = 57) I: One Step self-etch all bond universal ( *n* = 57)	USPHS Ryge modified	C:6 (10.52%) I:7 (12.28%)	Moisture control with cotton rolls; In patients with a single restoration, preference was given to intervention.	No statistically significant differences between groups. Fracture was the most common failure type.
Van Dijken and Pallesen (2015) 9 (Randomized Clinical Trial)	*N* = 158 C: Two-step etch-and-rise, Excite ( *n* = 69) I: One Step self-etch Xeno III ( *n* = 89)	USPHS Ryge modified	C: 21 (30.43%) I:26 (29.21%)	Moisture control with cotton rolls; In patients with a single restoration, preference was given to intervention.	No statistically significant differences between groups. Fracture was the most common failure type.
Van Dijken and Pallesen (2017) 25 (Randomized Clinical Trial)	*N* = 139 C: Three step TEGDMA/HEMA free etch-and-rise, CMF-els ( *n* = 70) I: One step HEMA free self-etch, Adhese One F ( *n* = 65)	USPHS Ryge modified	C:12 (17.14%) I:24 (36.92%)	Moisture control with cotton rolls; customized/modified adhesive.	The etch-and-rinse adhesive was better than the self-etch adhesive.
Çakir and Demirbuga (2019) 26 (Randomized Clinical Trial)	*N* = 133 C: Gluma Bond Universal, Clearfil Universal, Prime&Bond Elect Universal, All bond Universal and Single Bond Universal ( *n* = 99) I: Five step self-etch (Gluma Bond Universal, Clearfil Universal, Prime&Bond Elect Universal, All bond Universal and Single Bond Universal)( *n* = 100).	USPHS Ryge modified	C: 37 (37.38%) I: 29 (29%)	Used the same adhesives in the control and intervention groups, changing only the acid etching step.	No statistically significant differences between groups.
Perdigão et al (2009) 23 (Randomized Clinical Trial)	*N* = 199 C:Etch-and-rinse adhesive, One step Plus ( *n* = 23) I: Self-etching adhesives: iBond, ( *n* = 21), Clearfil SE ( *n* = 22) Adper Prompt ( *n* = 25)	USPHS Ryge modified	C: 6 (26.08%) I(Adper): 16 (64%) I(Clearfil): 11 (50.0%)	–	Control group resulted in statistically better good marginal adaptation than intervention groups. One intervention group (iBond) presented unacceptable outcome.

All studies compared etch-and-rinse (conventional) and self-etch adhesives with respect to the differences between failure rates of resin composite restorations in posterior teeth (Class I and II). All studies were randomized clinical trials. 9 23 24 25 26

A total of 699 resin composite restorations were analyzed during 2 23 to 8 9 years of follow-up; 342 restorations in the control group (etch-and-rinse adhesive), and 357 in the intervention group (self-etch). Only two studies 23 26 used rubber dam for moisture control during the restorative procedure.

The following brands of dentine adhesives were reported in the selected studies: Xeno III (Dentsply, Ballaigues, Suíça), Excite (Ivoclar Vivadent, Schaan, Liechtenstein), Prime&Bond Elect Universal (Dentsply, Milford, United States), Single Bond Universal (3M ESPE, Neuss, Germany), Gluma Bond Universal (Heraeus Kulzer, Germany), One Step Plus (Bisco, Schaumburg, United States), iBond (Heraeus Kulzer, Germany), Clearfil Universal Bond (Kuraray Noritake, Okayama, Japan), Clearfil SE (Kuraray Noritake, Okayama, Japan), Adper Prompt (3M ESPE, St Paul, United States), All-bond Universal (Bisco, Schaumburg, United States), OptiBond XTR (Kerr, Orange, United States) in addition to adhesives modified by the authors.

Generally, the studies used similar criteria of evaluation for **failed dental** restoration. The United States Public Health Services (USPHS) system was used, with some modifications among studies which did not preclude comparisons: USPHS Ryge system was reported in three articles, 9 24 25 while another paper 23 reported the Modified USPHS direct evaluation criteria. Calibrated examiners were reported in all papers. 9 23 24 25 26

From the quality assessment and risk of bias analysis using the Cochrane Collaboration Risk of Bias tool, two studies presented low risk, and the other three presented high risk ( Table 3 ). The main aspects related to high risk were modification of adhesives by authors, 25 and lack of use of rubber dam for moisture control. 9 24 25

**Table 3:** Quality assessment and risk of bias according to *Cochrane Risk of Bias Tool* 14

Studies	Sequence generation	Allocation Concealment	Blinding of participants and personnel	Blinding of outcome assessors	Incomplete outcome data	Selective outcome reporting	Other sources of bias	Risk of bias
Van Dijken and Pallesen (2017) 24	Yes	No	Yes	Yes	Yes	Yes	No	High risk
Van Dijken and Pallesen (2015) 9	Yes	No	Unclear	Yes	Yes	Yes	No	High risk
Van Dijken and Pallesen (2017) 25	Yes	No	Unclear	Yes	Yes	Yes	No	High risk
Çakir and Demirbuga (2019) 26	Unclear	No	Yes	Yes	No	No	No	Low risk
Perdigão et al (2009) 23	Unclear	Yes	No	Yes	Yes	No	Yes	Low risk

**Fig. 2:**
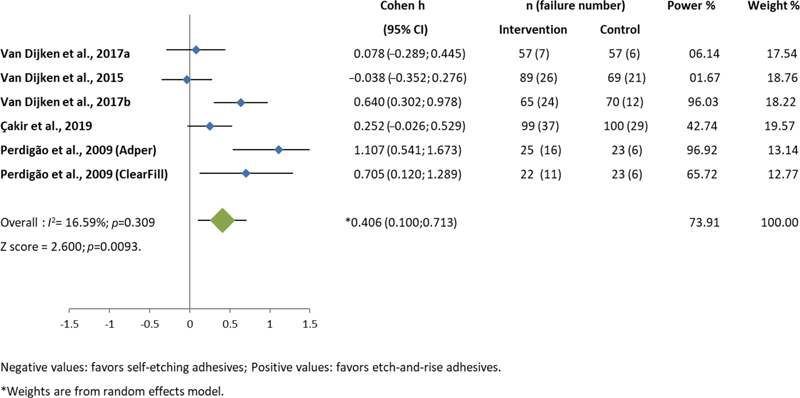
Results of meta-analyses: Negative Cohen H values favor self-etching adhesives. Positive Cohen H values favor etch-and-rise adhesives.

From the statistical analysis of individual studies, low statistical power was computed for most studies ( Fig. 2 ). The low power values are accompanied by wide 95% CIs of the effect size ranging from negative values (favoring the intervention group, self-etch adhesives) to positive values (favoring the control group, etch-and-rinse adhesives), indicating that sample sizes were smaller than required for the relatively large variability.

For each study, a single failure type accounted for the total number of restorations with failure: marginal adaptation in five studies 23 24 25 26 and marginal staining in one study. 9

Meta-analysis of all selected studies was performed using the random effects model due to the low heterogeneity ( *I* ^2^ = 16.59%; Cochrane Q test's *p* -value of 0.309) computed for this model. The meta-analysis showed a low summary positive effect size (0.406) with a wide 95% CI (0.100; 0.713; *p* = 0.0093) and power of 73.91%, favoring etch-and-rinse adhesives ( Fig. 2 ).

**Fig. 3:**
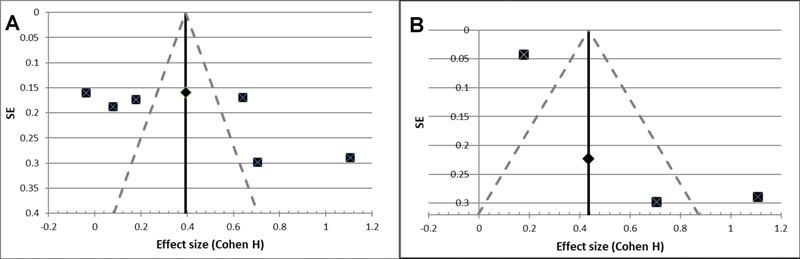
Funnel plot (Cohen H effect size against standard errors) of publication bias, for all studies.

The funnel plot detected the presence of important bias ( Fig. 3 ).

**Fig. 4:**
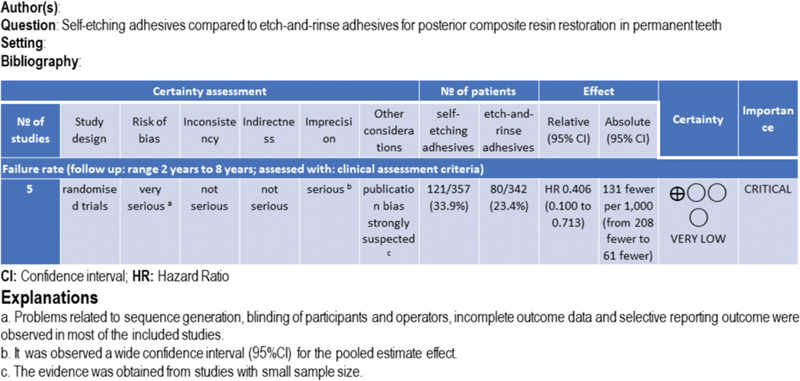
Grades of Recommendations, Assessment, Development and Evaluation (GRADE).

The assessment of the certainty of evidence through GRADE approach revealed a very low certainty of evidence ( Fig. 4 ). Although the certainty initiated as high through the five studies included in this systematic review, which were randomized controlled clinical trial, the process of detailed ratings across the five domains that can lower the certainty, downgraded this certainty. The critical domains were: (1) Risk of bias, illustrated in Table 3 , that revealed problems related to sequence generation, blinding of participants and operators, incomplete outcome data and selective reporting outcome in the majority of studies, leading to downgrading the certainty of evidence in two levels; (2) Imprecision, observed through the wide CIs. We downgraded the certainty just in one level, since the number of restorations included in metanalyses was above the rule-of-thumb of 400 (200 per group) and also above the optimal information size calculated ( *n* = 89 after loss of follow-up); (3) Publication bias, which was suspected analyzing the sample size of each study included, was small, and also confirmed through the funnel plot.

## Discussion

Our review detected six studies that met the inclusion criteria, and the main research question was whether the self-etch adhesives differed from etch-and-rinse adhesives in terms of failure rates of composite resin restoration in posterior permanent teeth. In addition to considering the statistical significance reported in the papers, we further computed effect sizes (intensity of the difference between failure rates), their CIs, and power. The analysis detected that four out of six selected studies presented individually wide CIs, which means that the sample size was smaller than required to yield reasonable standard errors. 10 11 When interpreting the high p-value and the wide 95% CI found for the meta-analysis of all studies (with and without rubber dam), one must consider that the probability within the CI is maximum at the point estimate (effect size of 0.403, favoring etch-and-rinse adhesives) and decreases towards both upper (0.703) and lower (0.100) limits. The words “were included” should be removed. 11 More specifically, the wide CI is the result of small sample sizes in the individual studies and can be improved by further studies with larger sample sizes. The presence of important publication bias ( Fig. 3 ) further supports the idea that the pooled studies have high variability.

The failures in marginal adaptation and marginal staining are closely related to the location of the dentine adhesive in the restoration, supporting the interpretation that the failure rate was mostly related to the adhesive type used. Self-etching adhesives face a coupled diffusion challenge: the outward diffusion of dissolved mineral ions (due to acid etching) and the inward diffusion of both the primer and the bonding molecules, with embedding of dissolved calcium phosphates within the dentin hybrid layer would destabilize the adhesive interface with time. 27 The lack of intermediate step potentially includes difficulties for establishing and reasonable hybrid layer, and the current recommendation includes a separate selective enamel acid conditioning prior to applying self-etching adhesives. 27 Such selective enamel conditioning was not used in any of the studies included in the current meta-analysis.

Our results are consistent with previous meta-analyses indicating higher sensitivity of self-etching adhesives to long-term water storage *in vitro* 28 and higher annual failure rates of one step self-etching adhesives in non-cervical carious lesions compared to both two steps etch-and-rinse and two steps self-etching adhesives. 29

The thinner hybrid layer obtained with self-etch adhesives 4 is another probable explanation for the higher failure rate of resin composite restoration in posterior teeth using self-etch adhesives.

In order to contribute to the planning of future longitudinal studies on the failure rates of etch-and-rinse versus one-step adhesives in posterior composite restorations, the use of rubber dam in paired groups recommended. For sample size calculations, to the best of available evidence identified in the current meta-analysis, it would be recommended the use of an effect size Cohen H of 0.406 (close to the cut-off of 5, for medium effect size), a two-tailed 5% significance level, power of 80%, which would result in a sample size of 48 per group. This estimation does not include any sample size loss due to the failure in compliance with study recall appointments during the follow-up period.

## Conclusion

In conclusion, current available evidence indicates that etch-and rinse adhesives performed better than self-etching adhesives in terms of failure rates in posterior composite restorations. But the certainty of evidence is very low, indicating the necessity of more well-conducted studies with larger sample sizes and less risk of bias. Improved ad hoc planning for future studies is required to achieve scientific evidence with smaller variability.
